# Sublingual Microcirculatory Characteristics of a Case of Profound Chemotherapy-Induced Anemia Treated With a Hemoglobin-Based Oxygen Carrier

**DOI:** 10.7759/cureus.15048

**Published:** 2021-05-15

**Authors:** Jason Stankiewicz, Maniraj Jeyaraju, Sanjay Maheshwari, Andrew R Deitchman, Michael T McCurdy

**Affiliations:** 1 Division of Pulmonary and Critical Care, University of Maryland School of Medicine, Baltimore, USA; 2 Critical Care Medicine, Christiana Care Health System, Newark, USA

**Keywords:** microcirculation, sublingual, anemia, hemoglobin-based oxygen carrier, resuscitation

## Abstract

Handheld vital microscopy (HVM) can deepen our understanding of hematologic diseases and therapeutics. However, limited reports have assessed human microcirculation during profound anemia, and response to hemoglobin-based oxygen carriers (HBOCs).

A 58-year-old woman presented with constitutional symptoms and was diagnosed with acute myeloblastic leukemia. Subsequently, the patient clinically decompensated and was found to have a hemoglobin of 1.9 g/dL. Human blood product administration was not consistent with her beliefs, and she received supportive care with HBOC-201. Concomitantly, her sublingual microcirculation revealed a markedly low microvascular flow index (2.59±0.26), proportion perfused vessels (66.8±18.8%), perfused vessel density (4.41±0.56 mm/mm^2^), and total vessel density (6.93±1.91 mm/mm^2^). HVM imaging is a promising point-of-care device for various hematologic conditions, with the potential to understand tissue-level perfusion in novel clinical scenarios, including profound anemia and HBOC administration, as illustrated in this case report.

## Introduction

Handheld vital microscopy (HVM) is an optical tool to analyze the microcirculation, a network of vessels less than 100 μm in diameter responsible for cellular level perfusion [1]. This technology can identify microcirculatory dysfunction despite normal macrocirculation parameters (e.g., blood pressure, cardiac output) in several disease states [2]. Current generation HVM devices, using incident dark-field (IDF) imaging, can measure microcirculatory parameters such as blood flow velocity, vessel density, and flow heterogeneity [2].

In this context, HVM devices deepen our understanding of hematological diseases and therapies, including various anemias and blood product administration. Although some reports have characterized the effects of anemia on human microcirculation [3-8], limited data have assessed the microcirculation during profound anemia. Moreover, no published literature describes the effects of hemoglobin-based oxygen carriers (HBOCs), such as HBOC-201 (Hemopure, Hemoglobin Oxygen Therapeutics, Souderton, PA), on human HVM parameters. Derived from polymerized bovine hemoglobin (Hb), HBOC-201 maintains the oxygen-carrying capacity of mammalian Hb while allowing for increased shelf life, negligible immune-mediated hemolysis, and support of anemic patients who decline human donor blood products [9].

We describe the lowest recorded Hb concentration (Hb 1.9 g/dL) in a patient for whom sublingual IDF-HVM imaging was used. Additionally, this is the first published assessment of HVM imaging characteristics in a patient receiving any HBOC product.

This article was previously presented as a meeting abstract at the 2020 American Thoracic Society International Conference in August 2020.

## Case presentation

A 54-year-old woman presented to the hospital for gait instability, lightheadedness, and fever. Her physical examination was notable for tachycardia (128 beats/minute) and a systolic flow murmur. Workup demonstrated normocytic anemia (hemoglobin (Hb) 5.7 g/dL), thrombocytopenia (platelet count 55x10^9^/L), hyperleukocytosis (white blood cell (WBC) 293x10^9^/L), and a peripheral smear demonstrating Auer rods with 96% blast cells. A computed tomographic (CT) scan of the chest revealed bilateral ground-glass opacities. The patient was admitted with a working diagnosis of new-onset acute myeloblastic leukemia complicated by neutropenic fever and community-acquired pneumonia.

While hospitalized, she received empiric antibiotics and supportive care that included prophylaxis against infection and tumor lysis syndrome. She underwent induction chemotherapy with peg-asparaginase, vincristine, and methylprednisolone. On hospital day 17, the patient’s blood cell counts progressively declined, she clinically decompensated, and was found to have a Hb of 1.9 g/dL. Concordant with her beliefs as a Jehovah’s Witness, she declined blood transfusion. She was treated with folic acid and erythropoietin, and phlebotomy was discontinued. She ultimately accepted HBOC-201 for a total of 3 units.

After HBOC administration, we documented the patient’s microcirculation using sublingual IDF-HVM [10]. CytoCam (Braedius Medical B.V., Huizen, The Netherlands) is an IDF-HVM device that utilizes a ring of light-emitting diodes (LED) at a wavelength of 530 nm released in a pulsed fashion of 2 milliseconds speed to illuminate the mucosa. Because Hb absorbs this light, it appears dark under IDF-HVM imaging, allowing for qualitative and quantitative analysis of the microvasculature. This device has a resolution of 0.66 μm/pixel, a field of view video of 1457x1061 μm, and improved usability compared to prior technologies [1]. A video clip is obtained by placing the device sublingually. The noninvasive location and relationship of the sublingual and systemic microcirculatory networks permit easy visualization [2].

**Figure 1:**
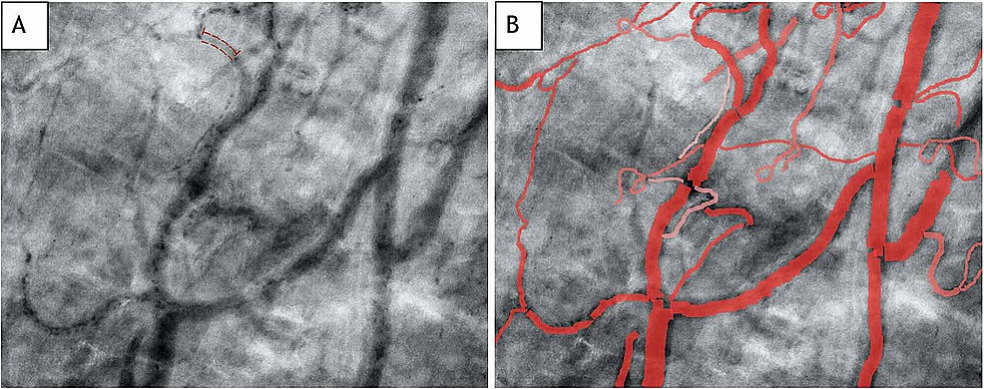
Representative sublingual handheld microscopy. Images obtained via the Cytocam device from the patient described in the text without the (A) and with the (B) annotation using Capillary Mapper 1.4.3. Image A demonstrates a relative paucity of red blood cells and the highlighted plasma gap indicative of dilutional or absolute anemia. Image B is annotated with outlined vessels correlating to TVD, and the intensity of each vessel correlates to an MFI score ranging from 0-3.

After the acquisition, each clip is stabilized using device software (CytoTools 1.7, Braedius Medical B.V.) and then analyzed by various software methods. We used the web-based Capillary Mapper software 1.4.3 tool for an offline, validated, semi-quantitative analysis [11]. Microcirculation parameters include two major categories: oxygen diffusion and convection [2]. Convection is measured by the microcirculatory flow index (MFI), which ranges from 0 to 3 (no flow (0), intermittent flow (1), sluggish flow (2), and normal continuous flow (3)) [1]. Diffusion is measured by total vessel density (TVD) (mm/mm^2^), perfused vessel density (PVD) (mm/mm^2^), and proportion of perfused vessels (PPV) parameters (%). Normal values in healthy volunteers using the Cytocam device for TVD, PVD, and PPV have been reported as 21.60±4.30 mm/mm^2^, 21.50±4.38 mm/mm^2^, and 100%, respectively [10]. A representative image of our patient’s microcirculation clips is demonstrated in Figure 1.

Qualitative analysis revealed a prominent space between individual red blood cells (RBCs) (i.e., plasma gap), indicating severe dilutional or absolute anemia. Semi-quantitative image analysis revealed a relatively low MFI score of 2.59±0.26. All markers of oxygen diffusion were markedly decreased, including TVD 6.93±1.91 mm/mm^2^, PVD 4.41±0.56 mm/mm^2^, and PPV 66.8±18.8%. Despite ongoing supportive care, the patient ultimately developed a spontaneous subarachnoid hemorrhage secondary to severe thrombocytopenia on hospital day 20 and she expired.

## Discussion

Several reports document sublingual HVM findings in subjects with various anemias. In the setting of hemodilution and hemorrhage, the microcirculation may exhibit a reduced density of normal-flow vessels, stagnant-flow vessels adjacent to normal-flow vessels, and hyperdynamic-flow vessels [2]. Other published reports have observed how microcirculation parameters respond to anemia and transfusions. Humans in hemorrhagic shock exhibited impaired microcirculation despite normal macrocirculation parameters, and this microcirculatory-macrocirculatory discordance portended a poor prognosis [2,8]. Adult post-cardiac surgery patients with anemia and hemodilution demonstrated increased sublingual microcirculation vessel density but not blood flow velocity 30 minutes after RBC administration [4]. Similarly, critically ill patients with hemorrhagic shock receiving RBCs had significantly increased MFI, PPV, and functional capillary density [7]. This correlation was also noted in hematology and oncology outpatients given RBCs for anemia, as they too exhibited improved sublingual microcirculation TVD without change in flow velocity [3].

Other reports suggest somewhat different observations. In a cohort of critically ill patients with severe sepsis, blood administration did not alter microcirculatory parameters. However, a specific subset of the population with lower baseline capillary perfusion had an improvement in vessel density and perfusion after RBC administration [5]. The observation that baseline, pre-transfusion microcirculatory measurements may impact the microcirculatory response to RBCs was also noted in surgical intensive care unit (ICU) patients with septic, cardiogenic, and/or hemorrhagic shock [12]. Another relationship between Hb concentration and microvascular density may exist, as evident in anemic pregnant patients (mean Hb 8.9 g/dL±0.8) who had higher TVD and PVD compared to healthy controls [6]. One proposed mechanism for increased vessel density in chronically anemic patients includes hypoxia-induced compensatory angiogenesis. Overall, these findings highlight the complex nature of the microcirculation during anemia, possibly resulting from the temporal course of anemia and the effects of underlying comorbid states (e.g., sepsis, trauma, myelosuppression) on the hemorheologic properties of microcirculation.

Our patient with life-threatening anemia was transfused with HBOC-201, a glutaraldehyde-based polymerized bovine Hb solution [9]. HBOC-201 has typically been clinically utilized when donor blood products are either unavailable or inconsistent with patient beliefs. However, limited animal models and no human studies have previously utilized sublingual HVM to characterize its effects on microcirculation. Because HBOC-201 absorbs light at the 340 nm, 415 nm, and 520-580 nm wavelength ranges, IDF-HVM sublingual imaging should be able to visualize it [13]. Therefore, one would expect HBOC-201 to improve the visualized microcirculatory parameters (e.g., vessel density) similar to Hb, however, HBOCs may induce other possible undesirable effects on the microvasculature as well.

Some concern exists that Hb, in its acellular form found in HBOC products, may induce microcirculatory vasoconstriction through the scavenging of nitric oxide by ferrous heme, which lowers available nitric oxide for vascular smooth muscle interaction. Song et al. utilized epi- and trans-illumination microscopy, a laboratory-based, non-handheld method to observe the microcirculation of the exteriorized spinotrapezius muscle of rats post-HBOC administration. They did not observe any changes in arteriolar diameter or macrovascular changes such as blood pressure [14]. Another study that administered HBOC-201 to anesthetized hamsters in hemorrhagic shock demonstrated improvements in trans-illumination microscopy functional vessel density and oxygen delivery but a decrease in microcirculation vessel diameters, suggesting possible vasoconstriction [15].

## Conclusions

In conclusion, we report sublingual microcirculation measurements in a patient with the lowest known value of Hb that has been measured with HVM imaging. Our patient’s measurements were generally consistent with reports documenting correlations between Hb concentration and microcirculation parameters. However, the magnitude of our patient’s microcirculatory dysfunction was more pronounced: numerous prominent plasma gaps were visualized, and quantitative measures (MFI, TVD, PVD, PPV) were markedly decreased. Additionally, we report the first case describing HVM imaging findings in a human receiving any HBOC. Although various animal-based and physiologic studies have evaluated the microcirculation after HBOC administration, further investigations to analyze its administration on the human sublingual microcirculation are necessary to better understand this relationship. HVM imaging is a promising point-of-care device to use on various hematologic disorders, with the potential to understand tissue-level perfusion in novel clinical scenarios such as profound anemia and the administration of HBOC products as illustrated in this unique case report.
